# Benign Pneumoperitoneum after Colonoscopy

**DOI:** 10.1155/2010/631036

**Published:** 2010-06-14

**Authors:** Sevim Ustek, Mertay Boran, Kemal Kismet

**Affiliations:** ^1^General Surgery Department, Cankiri State Hospital, 18100 Cankiri, Turkey; ^2^Thoracic Surgery Department, Cankiri State Hospital, 1810 Cankiri, Turkey; ^3^5th General Surgery Department, Ankara Training and Research Hospital, 06030 Ankara, Turkey

## Abstract

Pneumoperitoneum frequently indicates a perforated abdominal viscus that requires emergent surgical management. However; pneumoperitoneum, on rare occasion, can occur without perforation. In these cases, it is defined as benign pneumoperitoneum. Benign pneumoperitoneum means asymptomatic free intra-abdominal air or pneumoperitoneum without peritonitis and can occur occasionally with colonoscopy. In this paper, we present a rare case of benign pneumoperitoneum that developed after diagnostic colonoscopy and review it in conjunction with the current literature.

## 1. Introduction

Colonoscopy is a safe procedure with a low incidence of complication [1]. Colonic perforation resulting from colonoscopic procedures is rare but a serious complication with high rate of mortality and morbidity [2–5]. Benign pneumoperitoneum (BP), which can occur occasionally with colonoscopy, is defined as asymptomatic free intra-abdominal air or as pneumoperitoneum without peritonitis [1, 6, 7]. BP after diagnostic and therapeutic colonoscopy is rare, with an incidence at 0% to 3% [1]. Pneumoperitoneum detected after colonoscopy may pose a management dilemma [1, 8]. Symptomatic free air requires surgical management, but management of asymptomatic pneumoperitoneum is controversial [1]. If the etiology is microperforation, the standard treatment is intravenously administered antibiotics and bowel rest. However, transmural passage of insufflated air without bowel wall compromise may not require any intervention [1]. Conservative treatment should be reserved only for carefully selected patients [9]. 

We present and review this case in conjunction with the current literature because of its rarity and controversial treatment options.

## 2. Case Presentation

A 70-year-old male patient was admitted to emergency service with complaint of abdominal pain. He had a history of diagnostic colonoscopy performed 2 days before. Colonoscopy was a diagnostic procedure for evaluation of his complaints of right lower quadrant pain and constipation. Gastroenterologist that had performed the procedure indicated that the colonoscopic procedure was not complex and was performed safely. Room air was used for inflating the colon. Abdominal complaints of the patient started after colonoscopy and increased significantly. Abdominal pain, distention, and rigidity were detected on physical examination.

**Figure 1:**
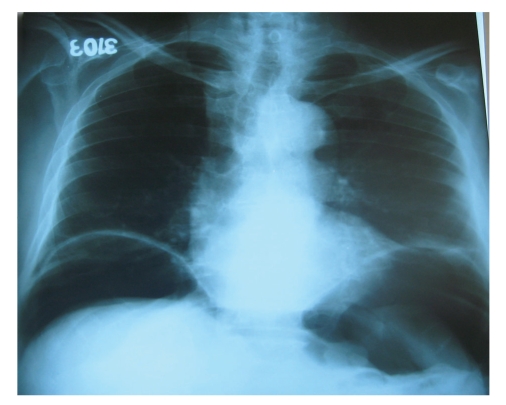
Chest radiography with free intra-abdominal air with elevated left and right hemidiaphragm.

**Figure 2:**
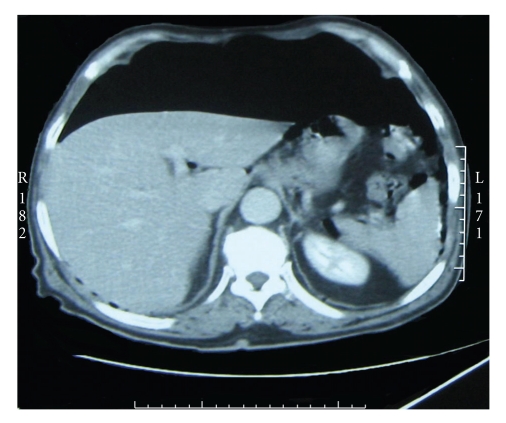
Abdomen CT with free intra-abdominal air.

 Laboratory findings were as follows: Leukocytes: 12000/mm^3^, Hb: 12 gr/dL, Hct: 35.4%, and CRP > 96 mg/L, fever: 37.8°C. Free air was detected on the plain films of the abdomen (Figure 1). On the abdominal computerized tomography, free air was detected (Figure 2). There was a 10 × 6 cm solid mass in the right iliac fossa. The border between the mass and iliac vein was not clear. There was another mass in the left iliac fossa 6.5 × 3 cm in dimensions. The prostate was hyperthrophic. 

The patient went under emergent operation. During the exploration, no perforation was detected in the gastrointestinal system. The mass in right iliac fossa was so fixed to the adjacent structures that, we could not remove this mass. The mass next to left iliac vein was unrelated to any organ in pelvis and it was removed completely. Abdomen was closed in layers. 

The postoperative course was uneventful. After the discharge of gas and stool, oral feeding was started in the postoperative 3rd day. No free air was seen in the plain film of the abdomen taken on postoperative 7th day and the patient was discharged from the hospital in health. The pathologic diagnosis of the mass was lymph node metastasis of adenocarcinoma. The result of the prostate biopsy taken in conjunction with transrectal ultrasonography was also adenocarcinoma. Upon these results, the patient was sent to medical oncology department for advanced treatment. Control tomography after oncologic treatment has not been performed yet.

## 3. Discussion

Colonoscopy is a safe procedure with a low incidence of complications and has great impact on diagnosis and management of diseases of the colon and rectum [1, 10]. Colonic perforation resulting from colonoscopic procedures is also rare. But it can cause serious complications with high rates of mortality and morbidity [2–5]. The frequency of perforations after colonoscopy is estimated to be 0.02% for diagnostic colonoscopy, and 0.09% for therapeutic (polypectomia) colonoscopy [11]. 

Pneumoperitoneum frequently indicates a perforated abdominal viscus that requires emergent surgical management because of visceral perforation in 85% to 95% of all cases [7]. Five to fifteen percent of the cases of pneumoperitoneum do not reflect perforation and result from another source that does not require emergent surgery [7].

BP is defined as asymptomatic free intra-abdominal air or pneumoperitoneum without peritonitis and appears as a characteristic radiolucency seen below the diaphragm on chest radiograph or in superiorly dependent location on abdominal radiograph [1, 7]. BP has been well described in various clinical scenarios besides colonoscopy, for example, after percutaneous endoscopic gastrostomy, laparotomy, or pneumatosis intestinalis [1]. BP after colonoscopy has been conjectured to occur more commonly after polypectomy or difficult studies, or transmural passage of insufflated air by using excessive insufflations [1, 7, 10].

Rare studies have prospectively investigated BP after colonoscopy [1, 11]. The vast majority of studies examining the complications of colonoscopy were retrospective [12, 13]. Therefore, all cases of pneumoperitoneum were discovered among symptomatic patients who had radiographs because of abdominal pain [1]. Pearl et al. [1] and Ecker et al. [11] conducted prospective studies and could not detect any benign pneumoperitoneum after colonoscopy. Therefore, our knowledge on benign pneumuperitoneum is limited to a few case reports [14, 15]. According to these reports, BP after diagnostic and therapeutic colonoscopy is rare, with an incidence at 0% to 3% [1].

Pneumoperitoneum detected after colonoscopy may pose a management dilemma [1, 8]. There are those who believe that all patients with a colon perforation following colonoscopy should have immediate surgery [16, 17]. Early laparotomy is thought to be associated with less morbidity and mortality [18]. Therefore, all cases of free intraabdominal air after colonoscopy have to be advocated as perforation [1]. However, management of intra-abdominal free air is various: Overt perforations necessitate laparotomy. When abdominal pain and distension are minimal, and peritoneal signs, fever and leukocytosis are absent, nonsurgical causes of pneumoperitoneum or microperforation have to be thought and these cases should be treated with intravenously administered antibiotics and bowel rest [1, 7, 9, 10, 12]. Transmural passage of air may not require treatment [1].

Although inflation of colon with CO_2_ may cause BP, this cannot be the reason of BP in our case; because we used room air for inflating colon during colonoscopy procedure.

Our patient had peritonitis and we performed laparotomy but there was no visible perforation. So, we decided that the reason of intraabdominal free air was transmural passage of air or microperforation, not a perforation. Then, we thought that the cause of peritonitis might be an intraabdominal tumor or metastasis. On exploration we found a mass next to the left iliac vein. Therefore, it should be kept in mind that some cases without perforation may be misdiagnosed as peritonitis. So, before the operation other factors which can cause pain and leukocytosis should be considered carefully.

In conclusion, the optimal treatment of pneumoperitoneum after colonoscopy, whether conservative or operative, is still unclear [9]. Until a large-scale study defines the incidence and treatment options, all cases of pneumoperitoneum after colonoscopy should be treated as perforation rather than innocuous transmural passage of air [1]. Therefore, patients with peritonitis are best treated by laparotomy and those with symptoms consistent with microperforation or no symptoms whatsoever might be treated with intravenous antibiotic therapy and bowel rest [1]. Conservative treatment should be reserved for only carefully selected patients [9].
